# Impacts of the Type I Toxin–Antitoxin System, SprG1/SprF1, on *Staphylococcus aureus* Gene Expression

**DOI:** 10.3390/genes12050770

**Published:** 2021-05-18

**Authors:** Kinga Chlebicka, Emilia Bonar, Piotr Suder, Emeline Ostyn, Brice Felden, Benedykt Wladyka, Marie-Laure Pinel-Marie

**Affiliations:** 1 Department of Analytical Biochemistry, Faculty of Biochemistry, Biophysics and Biotechnology, Jagiellonian University, 30-387 Krakow, Poland; chlebicka.kinga@gmail.com (K.C.); emilia.bonar@uj.edu.pl (E.B.); 2 Department of Analytical Chemistry and Biochemistry, Faculty of Materials Science and Ceramics, AGH University of Science and Technology, 31-007 Krakow, Poland; piotr.suder@agh.edu.pl; 3 Inserm, BRM [Bacterial Regulatory RNAs and Medicine]—UMR_S 1230, 35000 Rennes, France; emeline.ostyn@univ-rennes1.fr

**Keywords:** toxin–antitoxin systems (type I), *Staphylococcus aureus*, 2D-DIGE, proteomics, RNA antitoxin, peptide toxins

## Abstract

Type I toxin–antitoxin (TA) systems are widespread genetic modules in bacterial genomes. They express toxic peptides whose overexpression leads to growth arrest or cell death, whereas antitoxins regulate the expression of toxins, acting as labile antisense RNAs. The *Staphylococcus aureus* (*S. aureus*) genome contains and expresses several functional type I TA systems, but their biological functions remain unclear. Here, we addressed and challenged experimentally, by proteomics, if the type I TA system, the SprG1/SprF1 pair, influences the overall gene expression in *S. aureus*. Deleted and complemented *S. aureus* strains were analyzed for their proteomes, both intracellular and extracellular, during growth. Comparison of intracellular proteomes among the strains points to the SprF1 antitoxin as moderately downregulating protein expression. In the strain naturally expressing the SprG1 toxin, cytoplasmic proteins are excreted into the medium, but this is not due to unspecific cell leakages. Such a toxin-driven release of the cytoplasmic proteins may modulate the host inflammatory response that, in turn, could amplify the *S. aureus* infection spread.

## 1. Introduction

Toxin–antitoxin (TA) loci encode two-component modules that consist of a stable ‘toxin’, whose ectopic overexpression either kills cells or confers growth stasis, and an unstable ‘antitoxin’ that neutralizes the toxin action. A wealth of TA gene loci are present on bacterial plasmids, phages, and chromosomes [1]. These modules thrive in many bacterial pathogens, although the reasons for this profusion remain unknown. Despite studying TA modules for decades, their biological functions and underlying molecular mechanisms are lagging behind. Nevertheless, three functions were attributed to them, post-segregational killing after a plasmid loss, abortive infection, or persister cell formation. Thus, TA gene pairs act as effectors of dormancy and persistence and contribute to the generation of non-growing bacterial cells in response to stress, including during host cell internalization [2,3,4,5]. TA module expression regulation at the RNA and/or protein levels, toxin target selection and specificity, and antitoxin modes of negating the toxin effects, are elements that convert their actions into biological functions. Up to now, eight different TA system types were described [6] (type I to VI reviewed in [7]), but translational repression of toxin mRNA by an RNA antisense (type I) and inhibition of toxin activity by an antitoxin through protein–protein interaction (type II) are the prevailing mechanisms. Type I TA systems have an RNA antitoxin and a protein toxin. In these systems, antisense RNAs pair with the toxin’s mRNA to inhibit translation, and sometimes reduce a toxin’s mRNA steady-state levels [8]. Under standard growth conditions, those type I RNA duplexes cannot load onto the translating ribosomes and are rapidly degraded. Under stress, the pool of antitoxin RNAs is reduced, resulting in the translation of the toxin’s mRNA. The majority of type I toxins are short amphipathic peptides that can penetrate the membrane, disrupting its integrity and leading to growth arrest or cell death. Type I toxins are partitioned into two main classes, according to their locations and mechanisms. The first one comprises membrane-associated toxins acting by pore formation, by nucleoid condensation, or by forming a “carpet”, which destabilizes the phospholipid packing through a detergent-like mechanism. The second class is cytosolic toxins that cleave nucleic acids [9,10]. The *Staphylococcus aureus* N315, a multidrug-resistant pathogen, expresses functional type I TA systems [11,12,13,14,15]. Among them, the SprG/SprF TA systems are found in four homologs in the *S. aureus* genome [12,15]. The overexpression of toxic membrane peptides encoded by SprG RNAs triggers *S. aureus* stasis or death. SprF antitoxins reduce toxin mRNA steady-state levels by interacting in *cis* with their overlapping regions and thus prevent toxin translation [12,15]. The SprG toxicity control is specific to the cognate antitoxin, but cross-regulations between the different SprG/SprF TA systems can occur to modulate the RNA levels of homologs [15]. The biological function of the SprG1/SprF1 TA system begins to elucidate. The *sprG1*-encoded membrane peptides trigger lysis of competing bacteria and human cells [12]. Recently, we demonstrated that the SprF1 is a dual-function RNA antitoxin that, beyond protection from SprG1 toxicity, binds ribosomes to attenuate translation initiation by interfering with initiator transfer RNA binding. This translation attenuation mechanism mediated by SprF1 promotes antibiotic persister cell formation [16]. This may suggest a global regulatory role for the SprG1/SprF1 TA system, similar to already known staphylococcal gene expression regulators (such as *agr*, *sarA*, and *saeRS*) [17], albeit through completely different molecular mechanisms.

In this research, we performed a comparative proteomic study on the SprG1/SprF1 type I TA system to identify proteins, whose expression is modulated by SprF1 and/or SprG1 during *S. aureus* growth. We specifically monitored the differential expression of both intracellular and extracellular proteins in strain N315, a double deletion mutant, and an antitoxin-supplemented mutant. Comparison of intracellular proteomes among the strains points to the SprF1 antitoxin, as moderately downregulating protein expression. In the strain naturally expressing the SprG1 toxin, cytoplasmic proteins are excreted into the medium, but this is not due to unspecific cell protein leakage.

## 2. Materials and Methods

### 2.1. Bacterial Strains and Growth Conditions

*S. aureus* strain N315 [18] and the *sprG1/sprF1* deletion mutant (N315*ΔsprG1/ΔsprF1*) transformed with the control plasmid (pCN35), the N315*ΔsprG1/ΔsprF1* transformed with the plasmid (pCN35Ω*sprF1*) expressing SprF1 antitoxin under the control of its native promoter, as well as the N315*ΔsprG1/ΔsprF1* transformed with the plasmid (pCN35Ω*sprG1Flag*) expressing the flagged version of SprG1, under the control of its native promoter, [12] were plated or cultivated in Tryptic Soy Broth (TSB) supplemented with chloramphenicol (10 µg/mL) at 37 °C with vigorous shaking (200 rpm) (optimal conditions). For stress conditions, bacteria were grown either at elevated temperature (42 °C, TSB) or in minimal medium (NZM, 37 °C). For gene expression and proteomic analysis, cells were grown in optimal conditions with three and four biological replicates, respectively. When necessary, bacterial growth was monitored with a spectrophotometer, as optical density (OD) at 600 nm.

### 2.2. Proteomic Analysis

Sample preparation and 2D DIGE (two-dimensional difference gel electrophoresis) proteomic analysis were performed as described in [19]. Briefly, for cellular protein isolation, bacteria from the logarithmic and the stationary phase of growth (3.5 h and 15 h post-inoculation, respectively) were pelleted by centrifugation (5 min, 5000× *g*, 4 °C), washed twice with phosphate buffer saline, and suspended in TRI-Reagent (Sigma, Darmstadt, Germany). The cell suspension was homogenized (Precellys Evolution homogenizer, Bertin Technologies, Montigny-le-Bretonneux, France) 4× 30 s, 6300 rpm) and centrifuged (15 min, 10,000× *g*, 4 °C). The supernatant was collected, mixed with chloroform (4:1), and proteins were purified with the TRI-Reagent according to the manufacturer’s instructions for protein isolation and finally dissolved in a lysis buffer (7 M Urea, 2 M Thiourea, 30 mM Tris-HCl, 4% CHAPS). For extracellular protein isolation, the culture liquids were collected 16 h after inoculation with bacteria and centrifuged (30 min, 12× *g*, 4 °C). The obtained supernatants were filtered through a 0.22 µm PVDF filter to ensure the complete removal of bacterial cells. Proteins were precipitated with an equal volume of a mixture of methanol and chloroform (3:1), and the precipitate was dissolved in TRI-Reagent and proceeded as above.

For 2D DIGE, protein samples were labeled with fluorescent G-Dyes (Refraction-2D ™ Labeling Kits, NH DyeAGNOSTICS, Halle, Germany), paired, mixed respectively, and isoelectrofocused on 17 cm IPG-strips with a pH range of 4 to 7. SDS-PAGE was used for separation in the second dimension. The gels were then scanned with Chemi-doc MP (Bio-Rad, Hercules, CA, USA) and obtained images were analyzed with the DeCyder 7.2 software (GE Healthcare, Chicago, IL, USA). The differentiating protein spots were cut out from silver-stained gels and subjected to mass spectrometry identification. NanoLC-MS/MS analyses were done as described in [20] with minor changes. Briefly, after proteolytic cleavage with trypsin (Gold MS grade, Promega, Madison, WI, USA), samples were lyophilized and resuspended in 4% acetonitrile in water acidified by 0.1% formic acid (*v/v/v*). Next, peptides were introduced on the precolumn (300 µm ID × 10 mm), purified, and transferred on the capillary 75 µm ID × 150 mm column (both: C18, 5 µm, PepMap100, Thermo Fisher Scientific, Vilnius, Lithuania). The separation gradient was based on two solvents: water and acetonitrile, both acidified with 0.1% formic acid (*v/v*). The gradient parameters were 4 to 50% acetonitrile for 60 min. All gradients were performed using the Ultimate 3000 system (Thermo Scientific, Waltham, MA, USA) connected online to an AmaZon SL mass spectrometer (Bruker-Daltonics, Bremen, Germany). Basic settings of the mass spectrometer were as follows: scan range: 600 to 1800 m/z; ICC Target: 200,000 ions/ion trap cycle; spray voltage: 4200 V (source type: ESI nano sprayer); nebulizer pressure: 12 psi; gas flow: 5 L/min, heated capillary temperature: 140 °C. Fragmentation settings: precursor ions: 2; fragmentation scan range: 200 to 1800 m/z; preferred precursor charge: 2+; active exclusion of precursor mass envelope 4 Da after two fragmentation spectra for 30 s. Data extraction from raw files was done under Compass Data Analysis 4.4 SR1 (Bruker-Daltonics). The received *.mgf files were processed by Mascot software (www.matrixscience.com London, UK, database used: SwissProt, accessed on-line in the date range: October–December. 2020). The Mascot MS/MS ion search engine was set as follows: database: SwissProt, taxonomy: all entries; enzyme: trypsin (up to 1 missed cleavage); fixed modif.: carbamidomethylation; variable modif.: Met-oxidation; peptide tolerance ± 1.2 Da (13C = 1); MS/MS tolerance: ± 0.6 Da; peptide charge: 1+, 2+, 3+; instrument: ESI-TRAP. The proteomics data have been deposited to the ProteomeXchange Consortium via the PRIDE [21] partner repository with the dataset identifier PXD023449 and 10.6019/PXD023449. 

### 2.3. RNA Extraction, Northern Blots, and RT–qPCR Assays

The expression of the SprG1/SprF1 TA system components was monitored by Northern blot. *S. aureus* from logarithmic and stationary growth phases were centrifuged for 5 min at 4000× *g* at 4 °C and pellets were stored at −80 °C. Cell pellets were resuspended in 500 μL of lysis buffer (0.5% SDS, 20 mM sodium acetate, 1 mM EDTA, pH 5.5) and mechanically broken through bead beating in phenol (pH 4.0) using a FastPrep-24 5G instrument (MP Biomedicals, Illkirch-Graffenstaden, France). After 5-min centrifugation at 12,000× *g* at 4 °C, the aqueous phase was transferred with an equal volume of phenol (pH 4.0) and centrifuged for 5 min at 12,000× *g* at 4 °C. This was then mixed with an equal volume of a 24:1 solution of chloroform/isoamyl alcohol and centrifuged for 5 min at 12,000× *g* at 4 °C. RNA was precipitated from the aqueous phase by adding 2.5 volumes of ethanol and 0.1 volumes of 3 M NaOAc (pH 5.2) solution. For the Northern blot assays, 1 to 10 μg of RNA were separated onto an 8% urea–PAGE gel and electrotransferred onto a ZetaProbe GT membrane (Bio-Rad, Hercules, CA, USA) in 0.5× Tris-borate-EDTA buffer (90 mM Tris, 90 mM boric acid, 2 mM EDTA) for 2 h at 25 V. RNA was cross-linked to the membrane by ultraviolet irradiation. Specific probes (Table 1) were labeled with 0.5 μL [γ^32^P]-ATP (5 μCi; PerkinElmer, Waltham, MA, USA) and T4 PNK enzyme and hybridized overnight on the membranes with ExpressHyb solution (Ozyme, Saint-Cyr-l’École, France). Membranes were washed twice in 2× SSC solution with 0.05% SDS for 10 min, in 0.1× SSC with 0.1% SDS for 10 min, then exposed and scanned with a PhosphorImager (GE Healthcare, Chicago, IL, USA). The 5S ribosomal RNA was used as the loading control. mRNA levels of the SprG1 toxin and differentially expressed proteins were assessed by RT-qPCR. A total of 4 mL and 2 mL of bacterial cultures from logarithmic and stationary growth phases, respectively, were centrifuged (2 min, 5000× *g*, RT). Cells were resuspended in 1 mL of TRI-Reagent, transferred to the Lysis Matrix B Tube, and homogenized (Precellys Evolution homogenizer, Bertin Technologies, Montigny-le-Bretonneux, France, 4500 rpm, 15 min). Then, 100 μL of 1-bromo-3-chloropropan was added, the mixture was manually and vigorously shaken for 15 s and centrifuged (15 min, 12,000× *g*, 4 °C). The colorless upper phase was mixed with half a volume of 96% ethanol, transferred onto a microcolumn (Gene Jet RNA Purification Kit, Thermo Scientific, Dublin, Ireland), and spun (1 min, 12,000× *g*, RT). Next, 10 μL of DNase I RNase free (Thermo Scientific, Dublin, Ireland) was added to the column and incubated 15 min at RT. The next steps were performed according to Gene Jet RNA Purification Kit instructions. Total RNAs were eluted with water and reverse transcribed to cDNA by Maxima H Minus Reverse Transcriptase (Thermo Scientific, Dublin, Ireland), with random hexamers as primers. qPCR was performed with gene-specific primers (Table 1) and iTaq Universal SYBR Green Supermix (Bio-Rad, Hercules, CA, USA), with a 54 °C annealing temperature.

### 2.4. Protein Extraction and Western Blots

The *S. aureus* strains N315-transformed with pCN35 and N315*ΔsprG1/ΔsprF1* carrying either pCN35, pCN3535Ω*sprF1*, or pCN35Ω*sprG1Flag* plasmids were grown to the exponential and stationary growth phase in TSB. Protein samples were prepared from 10 mL of bacterial cultures. For intracellular protein extraction, bacterial pellets were resuspended in lysis buffer (50 mM Tris-EDTA, pH 7.7, 5 mM MgCl_2_, 100 μg ml^−1^ of lysostaphin), incubated for 15 min at 37 °C, then protease inhibitors (Roche Diagnostics, Meylan, France) were added and transferred onto ice. After sonication, protein concentration was quantified using the Qubit Protein Assay Kit (Thermo Scientific, Dublin, Ireland), according to the manufacturer’s instructions. For the extraction of extracellular proteins, supernatants were precipitated with 10% trichloroacetic acid overnight at 4 °C. After a 30-min centrifugation at 12,000× *g* at 4 °C, pellets were washed with 80% acetone and resuspended with 20 μL of loading buffer (37.5 mM Tris-HCl, pH 7, 0.75% SDS, 7.5% glycerol, 0.01% Coomassie Brilliant Blue, 5% β-mercaptoethanol). For the Western blots, 50 µg of intracellular protein extracts and all extracellular protein extracts were separated onto a 4 to 16% Tricine-SDS-PAGE gel [22]. Zinc staining (#161-0440, Bio-Rad, Hercules, CA, USA) was done as a loading control, then samples were transferred onto Amersham Hybond P polyvinylidene fluoride (PVDF) membranes (GE Healthcare, Chicago, IL, USA). After blocking with 5% bovine serum albumin in Tris-buffered saline, membranes were incubated (1 h at room temperature) with monoclonal mouse anti-Flag antibodies conjugated with horseradish peroxidase (1:2000, #A8592, Sigma, Darmstadt, Germany) then washed, and the signal was developed with an Amersham ECL Plus Western blotting Detection Kit and scanned with an ImageQuant LAS 4000 imager (GE Healthcare, Chicago, IL, USA). The presence of green fluorescent protein (GFP) inside and outside *S. aureus* cells was also monitored by Western blot. Briefly, N315 and N315*ΔsprG1/ΔsprF1* were transformed with pALCP2G expressing GFP under the control of a constitutive promoter [23]. Total cells from the stationary growth phase (mechanically disrupted with Precellys Evolution homogenizer, Montigny-le-Bretonneux, France) and the medium were separated using SDS-PAGE and transferred onto a PVDF membrane in CAPS buffer (10 mM CAPS pH 11.0, 10% methanol (*v/v*)). The membrane was blocked and incubated with primary anti-GFP antibodies (rabbit, 1:2000, #2555, Cell Signaling, Danvers, MA, USA), and then secondary antibodies (HRP-conjugated goat anti-rabbit, 1:40,000, #A6667, Sigma, Darmstadt, Germany). The signal was developed with chemiluminescence blotting substrate Immobilon Western HRP Substrate (Merck, Darmstadt, Germany).

### 2.5. In Vitro Transcription, RNA Labeling, and Electrophoretic Mobility Shift Assays (EMSA) Assays

SprF1 RNA and *ppiB_117_* mRNA were transcribed from PCR-amplified templates using N315 genomic DNA and forward primers containing a T7 promoter sequence (Table 1), using an Invitrogen MEGAscript T7 Transcription Kit (Thermo Scientific, Dublin, Ireland). according to the manufacturer’s protocol. RNA was purified on an 8% urea-PAGE gel and eluted overnight at 37 °C under shaking in 500 μL of elution buffer (20 mM Tris-HCl, pH 7.5, 250 mM NaCl, 1 mM EDTA, 1% SDS). The RNA was then purified by ethanol precipitation and pellets were resuspended with 20 μL of water and stored at −80 °C. For the labeling of the SprF1 RNA 5′-ends, RNA was dephosphorylated by CIP alkaline phosphatase (New England Biolabs, Ipswich, MA, USA) and labeled with 0.5 μL of [γ^32^P]-ATP (5 μCi) and T4 polynucleotide kinase (New England Biolabs Ipswich, MA, USA). Labeled SprF1 RNA was purified on Microspin ™ G-50 Columns (GE Healthcare, Chicago, IL, USA) and stored at −20 °C. Before use in EMSA assays, each transcribed RNA was denatured by incubation at 90 °C for 1 min, then chilled on ice for 1 min and refolded in a buffer (80 mM K-HEPES, pH 7.5, 4 mM MgCl_2_, 330 mM KCl). A total of 0.1 pmol of labeled SprF1 RNA was incubated with 6.25 to 100 pmol of *ppiB_117_* mRNA or 1 pmol of SprG1 RNA for 30 min at 30 °C. To evaluate specificity, 200 pmol of polyU RNA or 1 pmol of unlabeled SprF1 RNA were added. The samples were supplemented with glycerol (10% final concentration) and loaded on a native 8% polyacrylamide gel containing 5% glycerol. The electrophoresis was performed at 100 V in 0.5× Tris-borate EDTA at 4 °C. The results were analyzed on a PhosphorImager.

### 2.6. Statistics

Proteomic statistical analysis was performed based on a group-to-group comparison of Student’s *t*-test, and one-way ANOVA for multiple group comparison with a *p*-value < 0.05. The cutoff value for the average protein ratio was set to 1.5. RT-qPCR statistics were based on Student’s *t*-test.

## 3. Results

### 3.1. Deletion of the sprG1/sprF1 TA System Upregulates the Intracellular Proteome Whereas SprF1 Reverses It

**Figure 1:**
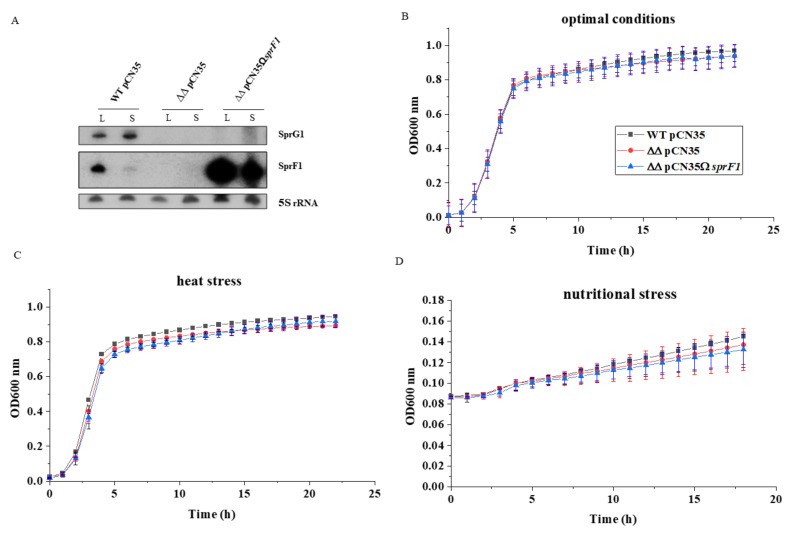
The SprG1/SprF1 type 1 TA system does not influence *S. aureus* N315 growth in laboratory conditions. (**A**) Northern blot analysis of the expression of SprG1/SprF1 system components in *S. aureus* N315 (WT pCN35), the *sprG1/sprF1* deletion mutant (ΔΔpCN35), and SprF1 antitoxin (over)expressing (ΔΔpCN35Ω*sprF1*) strains grown to logarithmic (L) and stationary (S) phases. (**B**–**D**) Growth curves in optimal and stress conditions.

To elucidate the impact of the SprG1/SprF1 TA system expression on the *S. aureus* proteome, we used the methicillin-resistant (MRSA) strain N315 and the mutant lacking the system. Moreover, the deletion mutant N315*ΔsprG1/ΔsprF1* was supplemented with a plasmid (pCN35Ω*sprF1*) expressing the SprF1 antitoxin. Northern blot analysis confirmed the presence/absence of transcripts for TA system components in the *S. aureus* respective strains at logarithmic (3.5 h post-inoculation) and stationary (15 h) growth phases. Moreover, the amount and relative proportions of transcripts for SprG1/SprF1 change over time. The antitoxin SprF1 decreases in the stationary growth phase, whereas the transcript for the SprG1 toxin does the opposite (Figure 1A). Deletion of *sprG1/sprF1*, as well as supplementation of the mutant with the antitoxin-encoding plasmid, did not impact bacterial growth in comparison to the isogenic strain. The same profile of growth curves was recorded for wild-type and mutant strains in optimal laboratory conditions (TSB, 37 °C), as well as in stress conditions such as elevated temperature (TSB, 42 °C) or nutritional stress (NZM, 37 °C) (Figure 1B–D).

**Figure 2:**
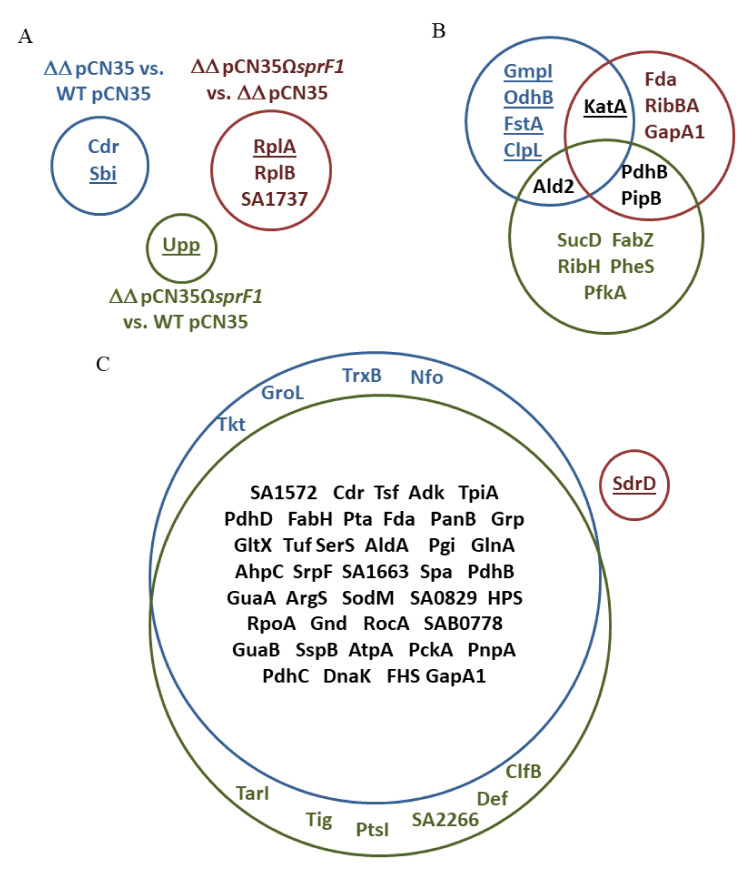
Impacts of the SprG1/SprF1 type I TA system on the *S. aureus* N315 proteome. Differentiating proteins in the intracellular proteome at logarithmic (**A**) and stationary (**B**) growth phases and in the extracellular proteome at stationary growth phase (**C**), comparing: wild-type (WT pCN35) and the SprG1/SprF1-deleted module (ΔΔ pCN35) (blue circle); WT pCN35 and SprF1 (over)expressing strain (ΔΔ pCN35ΩsprF1) (green circle); ΔΔ pCN35 and ΔΔ pCN35sprF1 (red circle). Proteins upregulated in comparison to the second component from the pair are underlined. For the meaning of the protein acronyms, please refer to Table 2 and Table 3.

To compare the intracellular proteomes, we collected cells from logarithmic and stationary growth phases. Cells were mechanically disrupted and proteins separated using 2D DIGE, followed by identification of differentially expressed proteins by mass spectrometry. We observed single differences in the proteomes of cells from logarithmic growth phase between the strains, in all three compared pairs (N315*ΔsprG1/ΔsprF1* vs. N315, N315*ΔsprG1/ΔsprF1* + pCN35Ω*sprF1* vs. N315, and N315*ΔsprG1/ΔsprF1* + pCN35Ω*sprF1* vs. N315*ΔsprG1/ΔsprF1*) (Table 2, Figure 2A, Supplementary Table S1). At the stationary growth phase, the differences in intracellular proteomes were more pronounced. In N315*ΔsprG1/ΔsprF1* vs. N315 pair, six differentiating proteins were identified, five of which were upregulated in the Δ*sprG1/*Δ*sprF1* mutant, indicating the role of the TA module in gene expression. Interestingly, however, in the strain with the enhanced expression of SprF1 antitoxin (N315*ΔsprG1/ΔsprF1* + pCN35Ω*sprF1*), eight differentiating proteins were identified, all downregulated, in comparison to the wild-type (N315) (Table 2, Figure 2B, Supplementary Figure S1). In N315*ΔsprG1/ΔsprF1* + pCN35Ω*sprF1* vs. N315*ΔsprG1/ΔsprF1* pair, five out of six differentiating proteins were downregulated in the strain with the enhanced expression of SprF1. This points to the antitoxin as the regulatory element of the SprG1/SprF1 TA system, as recently demonstrated [16].

**Figure 3:**
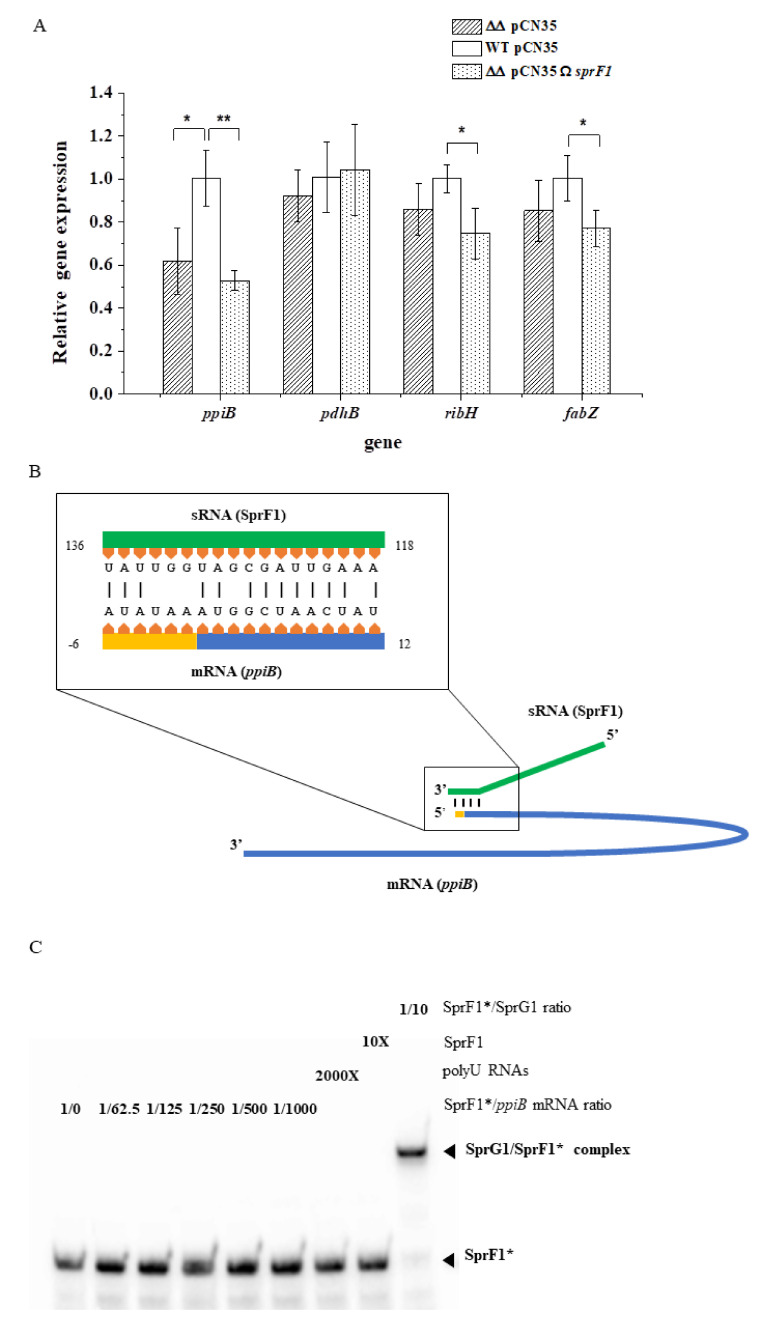
(Over)expression of SprF1 antitoxin decreases the level of gene transcripts expressing proteins identified as downregulated in the intracellular proteome. (**A**) RT-qPCR analysis of mRNAs isolated from *S. aureus* N315 (WT pCN35), *sprG1*/*sprF1* deletion mutant (ΔΔ pCN35), and the sprG1/sprF1 deletion mutant with the SprF1 antitoxin expressing (ΔΔ pCN35ΩsprF1) plasmid. * and **, denote statistical significance at *p* < 0.05 and *p* < 0.01 level, respectively. (**B**) A model of in silico predicted interactions between the gene transcript (*ppiB*) for peptidyl-prolyl cis-trans isomerase and the SprF1 antitoxin (sRNA). (**C**) EMSA of radioactively labeled SprF1 (SprF1 *) with mRNA for ppiB. SprG1/SprF1 * complex serves as a positive control.

Since type I antitoxins regulate the expression of the cognate toxin at the mRNA level [8], we were curious whether the same may be applied to the genes coding for proteins downregulated in the strain with the enhanced SprF1 expression. To this end, total RNAs were extracted from N315, N315*ΔsprG1/ΔsprF1*, and N315*ΔsprG1/ΔsprF1* + pCN35Ω*sprF1*, and RT-qPCR monitoring of the expression of four genes coding for proteins (3-hydroxyacyl-(acyl-carrier-protein) dehydratase (*fabZ*), 6,7-dimethyl-8-ribityllumazine synthase (*ribH*), pyruvate dehydrogenase E1 component subunit β (*pdhB*), and peptidyl-prolyl *cis-trans* isomerase (*ppiB*)), identified as the most downregulated in SprF1-supplemented strain, was performed. The amounts of *fabZ*, *ribH*, and *ppiB* transcripts were significantly decreased in the SprF1 overexpressing strain (Figure 3A), indicating that the regulation of the genes’ expression could also be detected at the mRNA level. However, the level of *ppiB* transcript was also decreased in the N315*ΔsprG1/ΔsprF1* mutant, pointing to the involvement of both components of the SprG1/SprF1 TA system in gene expression. Moreover, we searched for SprF1 targets in the *S. aureus* N315 genome, using TargetRNA2 [24]. Interestingly, among 23 predicted targets (Supplementary Table S2), we found the mRNA for PpiB, identified as substantially downregulated in the differential proteomic analysis. The predicted hybridization site of SprF1 with the gene transcript for PpiB covered the region directly downstream of the ribosome binding site and the start codon, which is the region for an effective gene expression silencing by *trans*-acting small RNAs [25] (Figure 3B). We performed electrophoretic mobility shift assays (EMSA) to analyze duplex formation between SprF1 and a 117 nt-long *ppiB* mRNA fragment containing the predicted site of interaction (58 nt downstream and 56 nt upstream of the AUG codon of *ppiB* mRNA). As a positive control, we showed a complex between SprF1 and SprG1 RNAs (Figure 3C, Pinel-Marie et al., 2014). However, no specific complex was observed between SprF1 and *ppiB_117_* mRNA (Figure 3C), indicating that the *ppiB* mRNA is not a direct target of SprF1. Altogether, these results allow us to carefully suggest that the SprF1 RNA antitoxin decreases the expression of eight intracellular proteins during the stationary growth phase, however, not as acting in *trans* with mRNA for downregulated proteins but rather by other indirect mechanisms.

### 3.2. The Lack of the SprG1 Toxin Correlates with Decreased Levels of Extracellular Proteins

**Figure 4:**
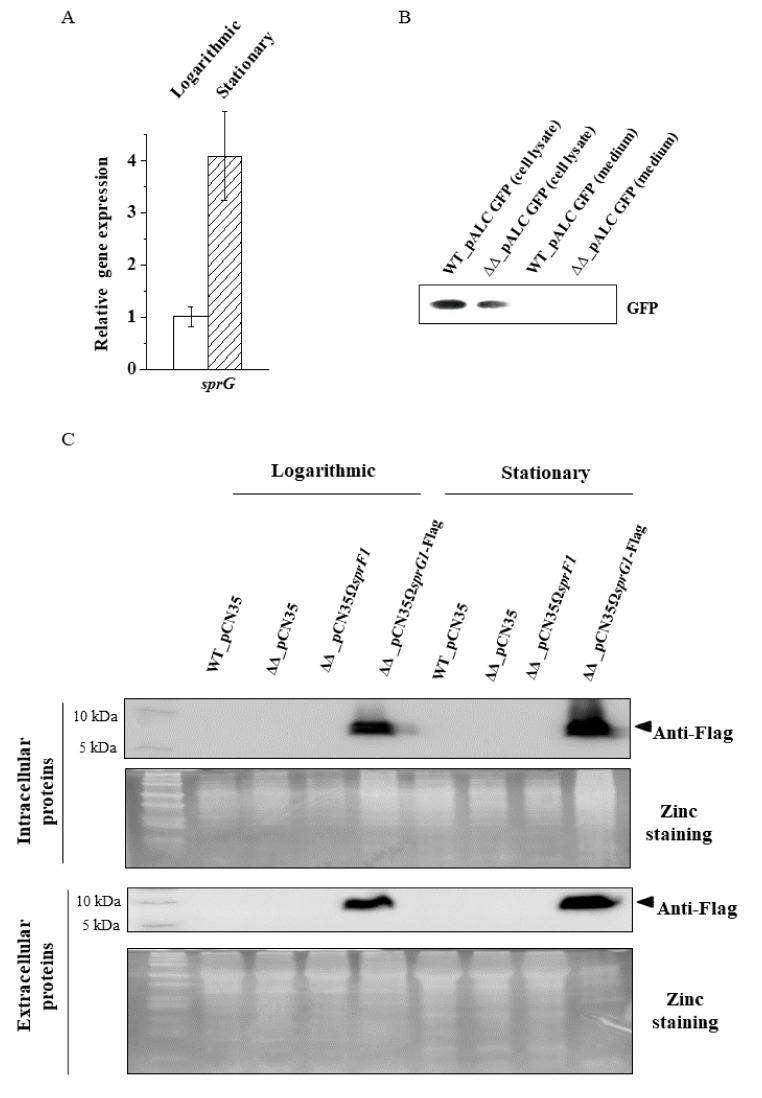
Expression of the SprG1-encoded peptides during the *S. aureus* stationary growth phase does not induce massive cell leakages. (**A**) RT-qPCR analysis of the transcript level for SprG1 toxin in *S. aureus* N315 (statistical significance at *p* < 0.01 level). (**B**) Western blot analysis with anti-GPF antibodies of cell lysates and spent growth medium of *S. aureus* N315 (WT) and the *sprG1/sprF1* deletion mutant (ΔΔ) transformed with a GFP-expressing plasmid (pALCP2G). (**C**) Western blot with anti-Flag antibodies and SDS-PAGE of total cell lysates (intracellular proteins; upper panels) and culture medium (extracellular proteins; lower panels) of *S. aureus* N315 (WT pCN35), the *sprG1/sprF1* deletion mutant (ΔΔ pCN35), SprF1 antitoxin (over)expressing (N315ΔΔ pCN35Ω*sprF1*), and Flagged SprG1 (over)expressing (N315ΔΔ pCN35Ω*sprG1-Flag*) strains grown to logarithmic and stationary phases.

Comparison of extracellular proteomes between the mutant strain without *sprG1/sprF1* TA system (N315*ΔsprG1/ΔsprF1*) and the wild-type strain (N315)*,* and between the *sprG1/sprF1* deletion mutant with the enhanced expression of SprF1 antitoxin (N315*ΔsprG1/ΔsprF1 + pCN35ΩsprF1*) and N315 resulted in the identification of 44 and 46 differentially expressed proteins, respectively (Table 3, Figure 2C, Supplementary Figure S2, and Supplementary Table S1). Strikingly, the expression of all those proteins was reduced in the *sprG1*/*sprF1* deletion mutants, compared to the isogenic N315 strain. Moreover, 37 proteins were common to both sets when compared. Interestingly, with the exception of four truly extracellular proteins (clumping factor B (ClfB), IgG-binding protein A (SpA), glutamyl endopeptidase (SspA) and cysteine protease, staphopain B (SspB)), and cell membrane-bounded ATP synthase subunit α (AtpA), all the other differentiating proteins were cytoplasmic. The presence of cytoplasmic proteins (CPs) in the exoproteomes has already been observed, but the mechanisms of their release are elusive [26]. We hypothesized that the presence of CPs outside the bacteria could reflect the activation of the SprG1/SprF1 type I TA system, resulting in the expression of the SprG1 toxin which, in turn, disrupts bacterial cell membranes and allows CPs to be expelled. One of the roles attributed to TA systems is to respond to environmental stress [6]. Transitions from logarithmic to stationary growth phases are a canonical example of a reaction to stress-induced by increasing cell density and exhaustion of nutrients. To test whether the SprG1/SprF1 TA system is involved in the response to such stress, we compared mRNA levels of the SprG1 toxin at logarithmic and stationary growth phases. RT-qPCR confirmed that the SprG1 mRNA level is over 3.5 higher in cells after a 15-h cultivation in comparison to those after 3.5 h post-inoculation (Figure 4A). The result is consistent with previous semi-quantitative Northern blot analysis (Figure 1A). Using the *S. aureus* strain overexpressing the flagged version of SprG1 (Supplementary Figure S3) [12] and performing Western blot using anti-Flag antibodies, we detected the presence of the *sprG1*-encoded peptide inside and outside the cell at the logarithmic and stationary growth phases (Figure 4C), suggesting that the SprG1 peptides permeabilize the *S. aureus* membrane to release CPs into the medium. To verify if the *sprG1*-encoded peptides can promote cell protein leakage by pore formation, as is the case for the TisB or HokB type I toxins [27,28,29], we transformed *S. aureus* N315 and the N315*ΔsprG1/ΔsprF1* mutant with a plasmid, pALCP2G, constitutively expressing GFP [23] and performed Western blots of post-culture medium with anti-GFP antibodies. Despite GFP overexpression in bacterial cells, we did not detect the presence of GFP in the medium of either strain (Figure 4B). This indicates that the release of CPs into the medium is not due to SprG1-driven massive cell leakage triggered by pore formation. Altogether, these results support the hypothesis that the SprG1-driven CP release could be related to a membrane disruption via a detergent-like effect or to an interference with membrane-associated functions, however, the elucidation of a specific mechanism requires further studies.

## 4. Discussion

In this study, we addressed and challenged experimentally, by proteomics, if the well-studied SprG1/SprF1 type I TA system influences gene expression in *S. aureus*. We adjusted our experimental approach accordingly by comparing the intracellular and extracellular proteome of *S. aureus* N315, naturally expressing the SprG1/SprF1 TA system, the TA knock-out strain (N315*ΔsprG1/ΔsprF1*) and the antitoxin complemented strain (N315*ΔsprG1/ΔsprF1* + pCN35Ω*sprF1*). Due to *sprG1*-encoded peptides toxicity, we could not obtain the strain that overexpresses only the SprG1 toxin [12]. Moreover, deleterious effects of toxins on the fate of bacterial cells were recently questioned, because the data was obtained from experimental set-ups, where the toxins were largely overexpressed [6,30], which is far from physiological conditions. We observed only single differences in intracellular proteome between wild-type, *sprG1*/*sprF1* deletion mutant and SprF1-complemented mutant, in the logarithmic phase of bacterial growth. This is consistent with the lack of differences in growth rates in optimal laboratory conditions (TSB, 37 °C), as well as in stress conditions (elevated temperature or nutritional stress) (Figure 1B–D). We showed that, at least in laboratory settings, the natural presence of the system does not impair bacterial growth and has a minor influence on the intracellular proteome during the intensive cell growth in comparison to the *sprG1/sprF1* deleted strain. However, at the stationary growth phase, the comparison of intracellular proteomes between the strains allows us to carefully suggest the SprF1 antitoxin as downregulating protein expression. We recently demonstrated that SprF1 is a dual function type I RNA antitoxin [16]. At its 3′-end, SprF1 acts as an antitoxin to prevent SprG1 toxicity towards competing bacteria and host cells [12]. Thanks to a purine-rich sequence located at its 5′-end, SprF1 also interacts with a subset of polysomes and ribosomes that could promote translation attenuation and antibiotic persister cell formation [16]. Among the proteins downregulated in the wild-type and the SprF1 antitoxin overexpressing strain or upregulated in the *sprG1/sprF1* deleted strain (Table 2), six are glycolysis-related enzymes: pyruvate dehydrogenase E1 component subunit β (PdhB), succinate-CoA ligase (ADP-forming) subunit α (SucD), ATP-dependent 6-phosphofructokinase (PfkA), 2,3-bisphosphoglycerate-independent phosphoglycerate mutase (GpmI), fructose-bisphosphate aldolase class 1 (Fda), and glyceraldehyde-3-phosphate dehydrogenase 1 (GapA1) (Supplementary Figure S4). Interestingly, the *pfkA*, *gmpI*, and *gapA1* transcript levels are downregulated in *S. aureus* intracellular antibiotic persisters [31]. These results may suggest the role of SprF1 in the *S. aureus* persister cell formation during the stationary growth phase, where the persister cell level is increased [32].

In type I TA systems, RNA antitoxins prevent the expression of cognate toxins at the mRNA level. We previously demonstrated that overexpression of SprF1 decreases mRNA for non-cognate toxins SprG2 and SprG3 [33]. We were curious as to whether SprF1 may have additional functions apart from silencing the expression of the SprG toxins, since we detected eight proteins downregulated in the SprF1 overexpressing strain at the stationary growth phase (Table 2). Interestingly, for the three proteins (6,7-dimethyl-8-ribityllumazine synthase, RibH; 3-hydroxyacyl-(acyl-carrier-protein) dehydratase, FabZ; peptidyl-prolyl cis/trans isomerase, PpiB) showing the highest downregulation by SprF1, the amount of the respective transcripts was significantly decreased in the SprF1 overexpressing strain, suggesting a direct interaction between the RNA antitoxin and these mRNAs (Figure 3A). In silico pairing predicted a possible interaction only between the *ppiB* transcript and SprF1. However, we did not see any complex between *ppiB* mRNA and SprF1 by EMSA assays (Figure 3C). PpiB (also referred to as PPIase) catalyzes the cis-trans isomerization of proline imidic peptide bonds in oligopeptides, thus accelerating protein folding. PPIases are required for the correct folding and subsequent activity of secreted virulence factors in a number of bacterial pathogens [34,35]. Another protein downregulated in *S. aureus* N315*ΔsprG1/ΔsprF1* + pCN35Ω*sprF1*, in the intra- and extracellular proteomes, is PdhB. The protein is a part of the pyruvate dehydrogenase complex that catalyzes the overall conversion of pyruvate to acetyl-CoA and carbon dioxide. Apart from its involvement in basic metabolism, PdhB is also found in the extracellular proteomes of many bacteria [36]. The moonlighting function for PdhB, as a fibronectin-binding protein facilitating host colonization and disease pathogenesis, was shown for the pathogen *Mycoplasma pneumonia* [37]. Although such a role in staphylococci was not confirmed experimentally, PdhB was reported as the only cellular protein belonging to the core exoproteome of the bovine *S. aureus* mastitis isolates [38]. Moreover, the intracellular proteins identified as downregulated by SprF1, especially PfkA and GapA1, were suggested in the *S. aureus* persister cell formation [16]. In general, the results suggest that the SprF1 RNA antitoxin influences the expression of some intracellular proteins. However, for transcripts of the proteins identified as downregulated in this proteomic study, SprF1 does not, most probably, serve as a *trans*-acting sRNA [25], which was exemplified for PpiB.

Analysis of extracellular proteomes indicated that the SprG1/SprF1-driven changes are noticeable. Among nearly 320 protein spots encompassing each exoproteome, we identified 44 and 46 differentiating (downregulated) proteins in N315*ΔsprG1/ΔsprF1* and N315*ΔsprG1/ΔsprF1* + pCN35Ω*sprF1* mutants, respectively. Thirty-seven of those proteins (over 80%) were common to both mutants (Figure 2C, Supplementary Figure S2). In contrast to the intracellular proteomes, only a single protein (serine-aspartate repeat-containing protein D, SdrD) differentiated exoproteomes of N315*ΔsprG1/ΔsprF1* and N315*ΔsprG1/ΔsprF1* + pCN35Ω*sprF1* mutants. Moreover, the SprF1 RNA level is decreased at the stationary growth phase, contrary to the SprG1 RNA level that is increased (Figure 1A and Figure 4A). This, together with the concise picture of exoproteomes in strains lacking the toxin, strongly suggests the role of SprG1 in the excretion of proteins outside the cell. Interestingly, the only differentially expressed *bona fide* secretory proteins in SprG1-devoid strains were cysteine protease staphopain B (SspB) and serine protease glutamylendopeptidase (SspA). The enzymes are part of the staphylococcal proteolytic system also consisting of metalloprotease (aureolysin) and other serine protease-like (SplA-F) proteases [39,40]. The enzymes are staphylococcal virulence factors that degrade host proteins and are involved in biofilm formation [41,42,43]. Moreover, two cell wall-anchored proteins, ClfB and SpA, were identified in the exoproteome. Both proteins bind host proteins, plasma fibrinogen, and type G immunoglobulin, respectively, and are important virulence factors (Supplementary Figure S4) [44]. The presence of cytoplasmic proteins (CPs) in the secretome was reported [26]. However, decreased amount of the proteins in *ΔsprG1/ΔsprF1* mutants points to the involvement of the TA system in their excretion. It was reported that overexpressed toxic peptides mimicking the activation of TA systems disrupt the cell membrane, leading to the release of the cell contents, which trigger cell death [12,14]. However, a natural level of SprG1 in *S. aureus* N315 does not induce unspecific protein leakage, as confirmed by Western blots with anti-GFP antibodies. The specific mechanism of excretion of CPs is unknown. However, in *S. aureus*, major autolysin (Atl) and α-type phenol soluble modulins (PSMα) both enhance the release of CPs outside the cell [45,46]. Moreover, excreted CPs contribute to the pathogenicity of *S. aureus*, since pathogenic strains excrete more CPs than non-pathogenic strains and species [45]. Interestingly, among proteins upregulated in the mutant strain was the cell division FtsA protein. The protein is involved in the assembly of the cytokinetic Z ring. The structure, among others, consists of polymers of the FtsZ protein, which is tethered to the cytoplasmic membrane at the division site by the C-terminal domain of FtsA [47]. Interestingly, the moment of cell division was suggested to be when cytoplasmic proteins are excreted outside the cell [46]. Moreover, FtsZ, the main component of the dividosome, is the molecular target for YeeU-YeeV, a type IV TA system in *E. coli* [48]. The SprG1/SprF1-driven regulation of FtsA suggests a role of the TA system in the decrease of *S. aureus* cell division during the stationary growth phase, where the availability of nutrients for bacteria is poor. We can speculate that SprG1-encoded peptides promote the release of the cytoplasmic proteins to the medium to favor staphylococcal growth, colonization, and infection when the environmental conditions become unfavorable.

## 5. Conclusions

In conclusion, in laboratory conditions, deletion of the SprG1/SprF1 type I TA system has no effect on *S. aureus* growth, however, it induces noticeable changes in the proteome. Some of the affected proteins are bona fide virulence factors or are involved in metabolic traits and have moonlighting functions that could be linked to staphylococcal pathogenicity [49]. Our proteomic data point to SprF1 RNA as a possible gene expression regulator. However, the number and fold changes of differentially expressed proteins do not allow us to unambiguously support the role of the antitoxin in the *S. aureus* translation attenuation by binding ribosomes, a role that our recent study linked with persister cell formation [16]. Nevertheless, the above findings demonstrate a general function for bacterial RNA antitoxin beyond protection from toxicity. We also speculate about the role of the SprG1 toxin in the release of the cytoplasmic proteins. These observations require further studies to identify the mechanism of action of the *sprG1*-encoded peptides and to elucidate the roles of the SprG1/SprF1 module in staphylococcal pathogenicity, including virulence and persister cell formation.

## Figures and Tables

**Table 1:** A list of DNA oligonucleotides used in the study.

Name	Sequence (5′-3′)	Application
SprF1-NB	TAACTTTGGCTGGTTTCGATGGTT	Northern blot
SprG1-NB	ATGCCACCATAGGCACCACCTCCTT	Northern blot
5S rRNA-NB	CGTAAGTTCGACTACCATCG	Northern blot
SprG1-F	AGTATACAAGCAGTAAAAAAAGTATATGTG	RT-qPCR
SprG1-R	ATTTCAGTAATGCCACCATAGGCA	RT-qPCR
gyrB-F	CAACAATGAACCCTGAGCACC	RT-qPCR
gyrB-R	CGGTTTTCTACAACGTCACCC	RT-qPCR
ribH-F	GTCGCGAAAGGTGTTTCTAAAGTA	RT-qPCR
ribH-R	CCAGCTTTCGTACCTGCTCT	RT-qPCR
fabZ-F	AACGTCAAGTAGTACCTGGTGATA	RT-qPCR
fabZ-R	CAAGCAAGTTGACCATCGACAG	RT-qPCR
ppiB-F	CATTGTTCAAATGAAAGAAGTACCTCA	RT-qPCR
ppiB-R	GTGTACCACCCTTTTCGCCATA	RT-qPCR
pdhB-F	GCTGAATCAGGTATTGGTGGTTTA	RT-qPCR
pdhB-R	TGTCCAGCAATCGCATCAAATACTT	RT-qPCR
SprF1-T7	TAATACGACTCACTATAGGGATATATAGAAAAAGGGCAAC	In vitro transcription
SprF1-rev	AAAAAATAACCATCGCTAACTTTGGCT	In vitro transcription
ppiB-T7	TAATACGACTCACTATAGGGTTTCCTCCCTTAAAAGTATGTTAATA	In vitro transcription
ppiB-rev	ATAACCACTTTAATTTCACCTTGTT	In vitro transcription

**Table 2:** List of differentially expressed proteins in the comparison of the intracellular proteome of *S. aureus* N315 with either the deletion of the SprG1/SprF1 TA system (*ΔΔ*) or with the (over)expression of the SprF1 antitoxin (*ΔΔSprF1*).

No.	Protein (Acronym)	Accession Number	N315-*ΔΔ* vs. N315	N315-*ΔΔSprF1* vs. N315	N315-*ΔΔSprF1* vs. N315-*ΔΔ*
**Logarithmic phase**					
1	Coenzyme A disulfide reductase (Cdr)	Q7A6H1	1.68 ↓ *		
2	Immunoglobulin-binding protein (Sbi)	Q99RL2	1.72 ↑		
3	50S ribosomal protein L1 (RplA)	Q99W68			1.82 ↑
4	50S ribosomal protein L2 (RplB)	P60432			1.51 ↓
5	Uncharacterized protein (SA1737)	Q7A4P4			1.50 ↓
6	Uracil phosphoribosyltransferase (Upp)	P67396		2.19 ↑	
**Stationary phase**					
1	Pyruvate dehydrogenase E1 component subunit β (PdhB)	P99063		2.19 ↓	1.82 ↓
2	Succinate--CoA ligase (ADP-forming) subunit α (SucD)	P99070		1.55 ↓	
3	Putative peptidyl-prolyl cis-trans isomerase (PpiB)	Q7A6I1		2.19 ↓	1.82 ↓
4	3-hydroxyacyl-(acyl-carrier-protein) dehydrataseFabZ (FabZ)	P64108		2.36 ↓	
5	6,7-dimethyl-8-ribityllumazine synthase (RibH)	P99141	1.58 ↓	2.36 ↓	
6	Alanine dehydrogenase 2 (Ald2)	Q99TF4		1.86 ↓	
7	Phenylalanine--tRNA ligase α subunit (PheS)	P68848		1.55 ↓	
8	ATP-dependent 6-phosphofructokinase (PfkA)	P99165		1.55 ↓	
9	2,3-bisphosphoglycerate-independent phosphoglycerate mutase (GpmI)	P64270	1.53 ↑		
10	Dihydrolipoyllysine-residue succinyltransferase component of 2-oxoglutarate dehydrogenase complex (OdhB)	Q7A5N4	1.53 ↑		
11	Catalase (KatA)	Q7A5T2	1.66 ↑		1.90 ↑
12	Cell division protein FtsA (FtsA)	P63765	1.53 ↑		
13	ATP-dependent Clp protease ATP-binding subunit ClpL (ClpL)	Q7A3F4	1.53 ↑		
14	Fructose-bisphosphate aldolase class 1 (Fda)	P99117			1.54 ↓
15	Riboflavin biosynthesis protein RibBA (RibBA)	Q7A511			1.62 ↓
16	Glyceraldehyde-3-phosphate dehydrogenase 1 (GapA1)	P99136			1.62 ↓

* the arrow denotes the direction of regulation; ↑ up- and ↓ down-regulation in comparison to the second component from the pair, respectively.

**Table 3:** List of differentially expressed proteins from the extracellular proteome in comparing *S. aureus* N315 and its mutants with the deletion of SprG1/SprF1 TA system (*ΔΔ*) and with the (over)expression of SprF1 antitoxin (*ΔΔSprF1*).

No.	Protein (Acronym)	Accession Number	N315-*ΔΔ* vs. N315	N315-*ΔΔSprF1* vs. N315	N315-*ΔΔSprF1* vs. N315-*ΔΔ*
1	Glutamate----tRNA ligase (GltX)	P99170	1.74 ↓	1.72 ↓	
2	1-pyrroline-5-carboxylate dehydrogenase (RocA)	P99076	1.96 ↓	2.18 ↓	
3	Glutamine synthetase (GlnA)	P99095	1.77 ↓	2.12 ↓	
4	GMP synthase [glutamine-hydrolyzing] (GuaA)	P99105	1.87 ↓	1.80 ↓	
5	Arginine--tRNA ligase (ArgS)	Q99W05	1.87 ↓	1.80 ↓	
6	Transketolase (Tkt)	P99161	1.67 ↓		
7	6-phosphogluconate dehydrogenase, decarboxylating (Gnd)	P63334	1.94 ↓	1.85 ↓	
8	Phosphoenolpyruvate carboxykinase (ATP) (PckA)	P99128	2.57 ↓	2.42 ↓	
9	Glucose-6-phosphate isomerase (Pgi)	P99078	1.77 ↓	1.86 ↓	
10	Dihydrolipoyllysine-residue acetyltransferase component of pyruvate dehydrogenase complex (PdhC)	P65636	2.71 ↓	2.00 ↓	
11	Pyruvate dehydrogenase E1 component subunit β (PdhB)	P99063	1.84 ↓	2.23 ↓	
12	Dihydrolipoyl dehydrogenase (PdhD)	P99084	1.66 ↓	1.60 ↓	
13	Fructose-bisphosphate aldolase class 1 (Fda)	P99117	1.68 ↓	1.56 ↓	
14	Triosephosphate isomerase (TpiA)	P99133	1.59 ↓	1.92 ↓	
15	Glyceraldehyde-3-phosphate dehydrogenase 1 (GapA1)	P99136		1.5 ↓	
16	3-hexulose-6-phosphate synthase (HPS)	Q7A774	1.92 ↓	2.11 ↓	
17	Formate--tetrahydrofolate ligase (FHS)	Q7A535	3.41 ↓	2.75 ↓	
18	Phosphoenolpyruvate-protein phosphotransferase (PtsI)	Q99V14		1.63 ↓	
19	Elongation factor Ts (Tsf)	P99171	1.59 ↓	1.54 ↓	
20	Elongation factor Tu (Tuf)	P99152	1.74 ↓	1.93 ↓	
21	Serine--tRNA ligase (SerS)	P99178	1.74 ↓	2.12 ↓	
22	Chaperone protein DnaK (DnaK)	P99110	1.82 ↓	1.64 ↓	
23	Protein GrpE (GrpE)	P99086	1.73 ↓	1.50 ↓	
24	Trigger factor (Tig)	P99080		1.54 ↓	
25	60 kDa chaperonin (GroL)	P99083	1.51 ↓		
26	Thioredoxin reductase (TrxB)	Q6GIM7	2.02 ↓		
27	Alkyl hydroperoxide reductase C (AhpC)	P99074	1.82 ↓	1.82 ↓	
28	Superoxide dismutase [Mn/Fe] 2 (SodM)	P66831	1.90 ↓	1.73 ↓	
29	Coenzyme A disulfide reductase (Cdr)	Q7A6H1	1.54 ↓	1.60 ↓	
30	Glutamyl endopeptidase (SspA)	Q7A6A6	1.80 ↓		
31	Immunoglobulin G-binding protein A (SpA)	P99134	1.83 ↓	1.84 ↓	
32	Staphopain B (SspB)	Q7A6A7	2.27 ↓	1.81 ↓	
33	Clumping factor B (ClfB)	Q7A382		1.63 ↓	
34	ATP synthase subunit α (AtpA)	P99111	2.42 ↓	2.41 ↓	
35	Adenylate kinase (Adk)	P99062	1.59 ↓	1.59 ↓	
36	Inosine-5’-monophosphate dehydrogenase (GuaB)	P99106	2.06 ↓	1.95 ↓	
37	Polyribonucleotide nucleotidyltransferase (PnpA)	Q7A5 × 7	2.71 ↓	1.99 ↓	
38	Probable endonuclease 4 (Nfo)	P63538	1.65 ↓		
39	DNA-directed RNA polymerase subunit α (RpoA)	P66706	1.93 ↓	1.81 ↓	
40	Peptide deformylase (Def)	P99077		1.82 ↓	
41	Ribitol-5-phosphate cytidylyltransferase 1 (TarI)	Q7A7V0		2.01 ↓	
42	3-oxoacyl-(acyl-carrier-protein) synthase 3 (FabH)	P99159	1.66 ↓	1.68 ↓	
43	Phosphate acetyltransferase (Pta)	P99092	1.66 ↓	1.96 ↓	
44	UPF0051 protein (SAB0778)	Q7A6L4	1.97 ↓	1.96 ↓	
45	3-methyl-2-oxobutanoate hydroxymethyltransferase (PanB)	P65656	1.71 ↓	1.89 ↓	
46	DUF4242 domain-containing protein (SA0165)	A0A0H3JSJ2		1.58 ↓	
47	Putative aldehyde dehydrogenase (AldA)	Q7A825	1.74 ↓	2.12 ↓	
48	Putative dipeptidase (SA1572)	Q7A522	1.51 ↓	2.32 ↓	
49	Uncharacterized oxidoreductase (SA2266)	Q7A3L9		2.52 ↓	
50	Uncharacterized protein (SA0829)	Q7A6H3	1.90 ↓	1.79 ↓	
51	UPF0342 protein (SA1663)	Q7A4V3	1.82 ↓	2.17 ↓	
52	Serine-aspartate repeat-containing protein D (SdrD)	Q7A780			1.58 ↑

* the arrow denotes the direction of regulation; ↑ up- and ↓ down-regulation in comparison to the second component from the pair, respectively.

## Data Availability

The proteomics data have been deposited to the ProteomeXchange Consortium via the PRIDE partner repository with the dataset identifier PXD023449 and 10.6019/PXD023449.

## Supplementary Materials

The following are available online at https://www.mdpi.com/article/10.3390/genes12050770/s1, Figures S1 and S2: An exemplary 2D DIGE gel of intracellular and extracellular proteins, respectively, of *S. aureus* N315 and the SprG1/SprF1 TA system deletion mutant transformed with a plasmid (over)expressing the SprF1 antitoxin isolated from stationary growth phase, Figure S3: Growth curve of *S. aureus* N315 *sprG1/sprF1* deletion mutant transformed with the plasmid (pCN35Ω*sprG1-*FLAG) expressing the flagged version of SprG1 toxin, cultivated in optimal conditions, Figure S4: Voronoi diagrams illustrating functions of differentially expressed proteins identified in proteomic studies, Table S1: A list of differentially expressed proteins extracted from proteomic comparisons of *S. aureus* N315 with its deletion mutant of SprG1/SprF1 TA system and with the (over)expression of SprF1 antitoxin, Table S2: SprF1 (sRNA) interactions with *S. aureus* N315 gene transcript predicted by the TargetRNA2 algorithm.
