# Supplementary material for: Impacts of the Type I Toxin–Antitoxin System, SprG1/SprF1, on Staphylococcus aureus Gene Expression
Source: Genes (Basel). 2021 May 18;12(5):770. doi: 10.3390/genes12050770 (PMC8158120; doi:10.3390/genes12050770)
Supplement: Supplementary file 1 [file genes-12-00770-s001.zip › Supplementary Data Genes rev.pdf]

Supplementary data for

Impacts of the type I toxin-antitoxin system, SprG1/SprF1, on *Staphylococcus aureus* gene expression

Kinga Chlebicka, Emilia Bonar, Piotr Suder, Emeline Ostyn, Brice Felden, Benedykt Wladyka and Marie-Laure Pinel-Marie

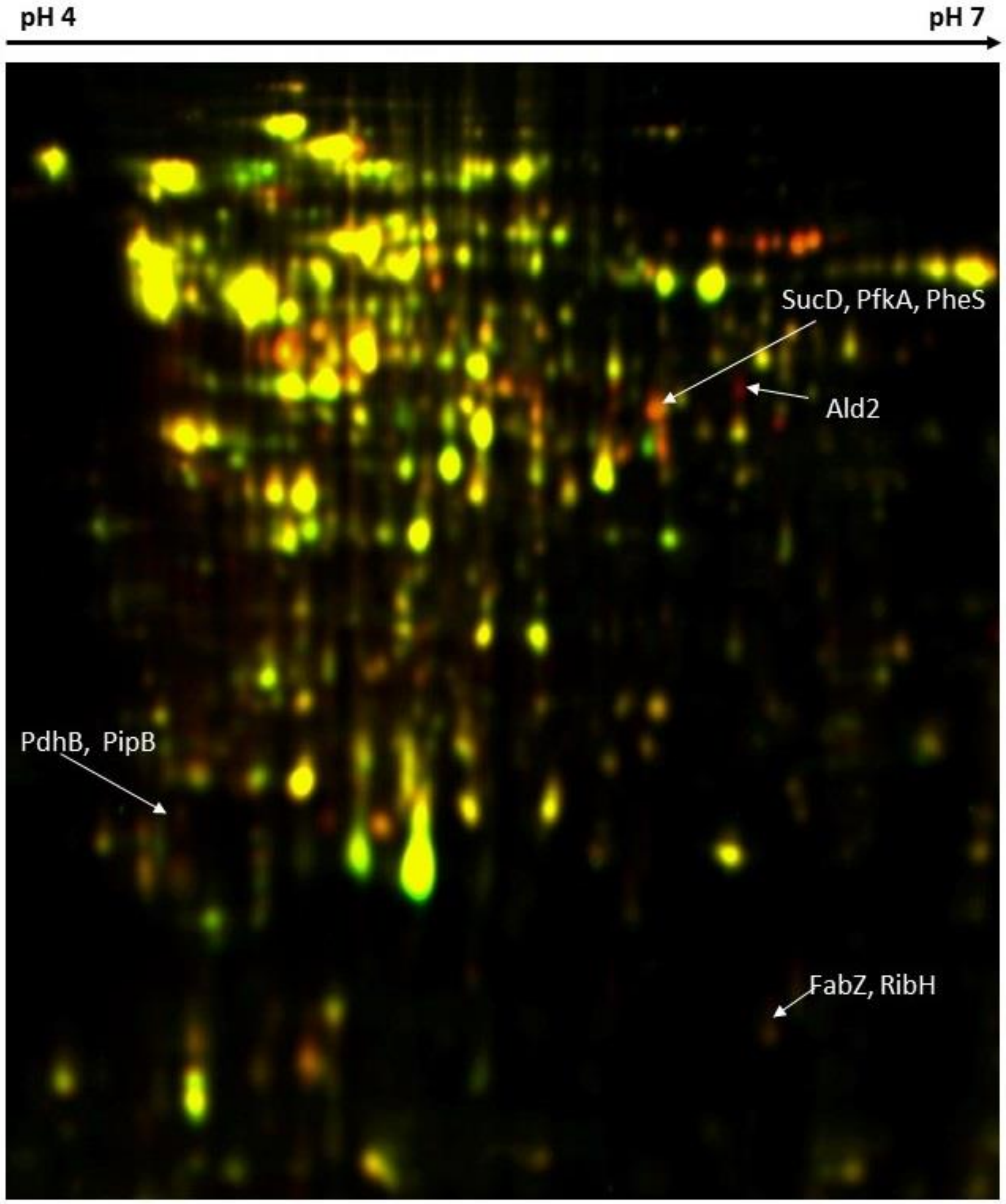

Supplementary Figure S1. An exemplary 2D DIGE gel of *S. aureus* N315 and the SprG1/SprF1 TA system deletion mutant, transformed with a plasmid (over)expressing the SprF1 antitoxin, intracellular proteins isolated from stationary growth phase (15 h). Differentiating protein spots are marked with arrows. For the meaning of the protein acronyms, please refer to Table 2.

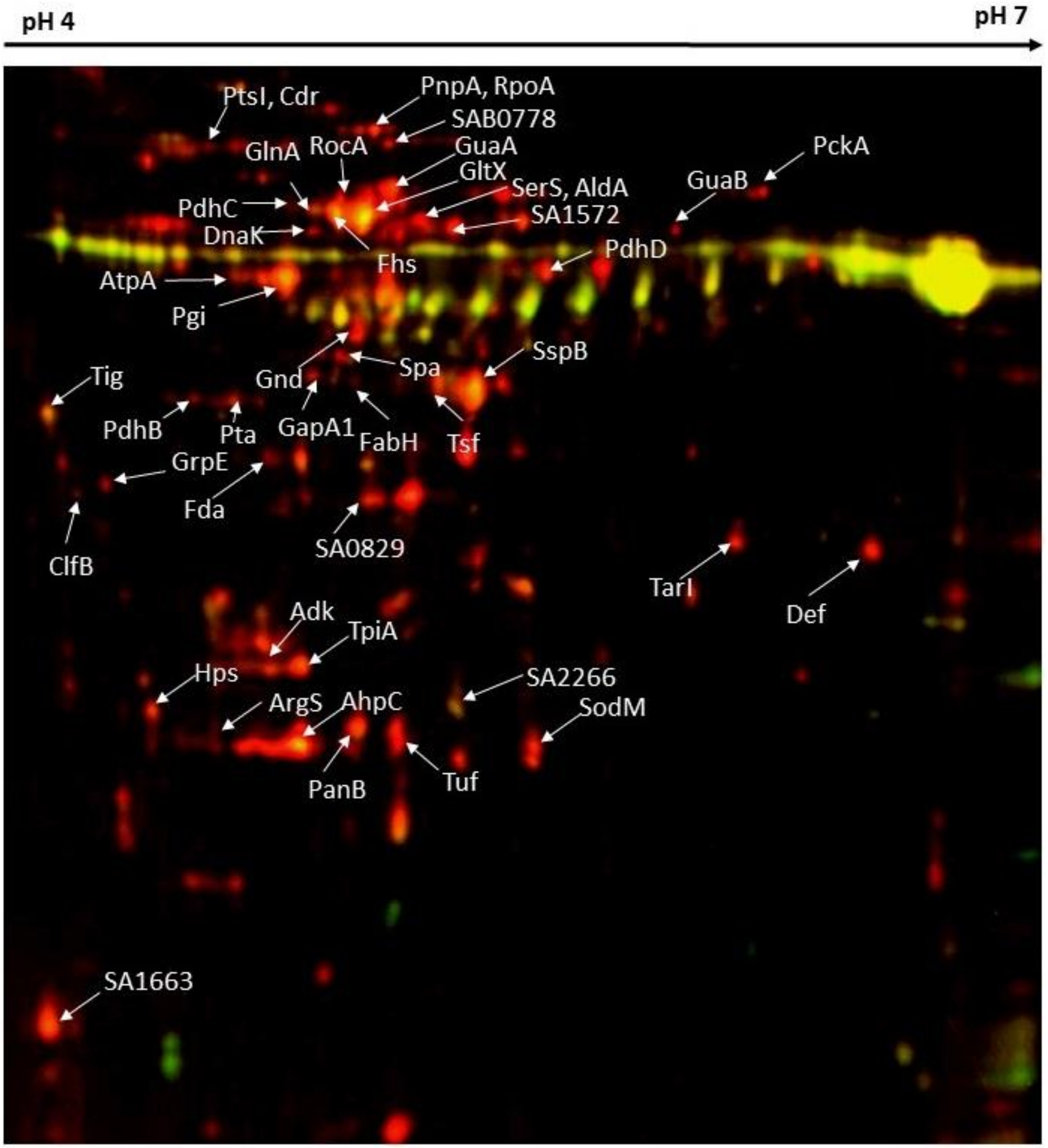

Supplementary Figure S2. An exemplary 2D DIGE gel of *S. aureus* N315 and the SprG1/SprF1 TA system deletion mutant, transformed with a plasmid (over)expressing the SprF1 antitoxin, extracellular proteins isolated from stationary growth phase (15 h). Differentiating protein spots are marked with arrows. For the meaning of the protein acronyms, please refer to Table 3.

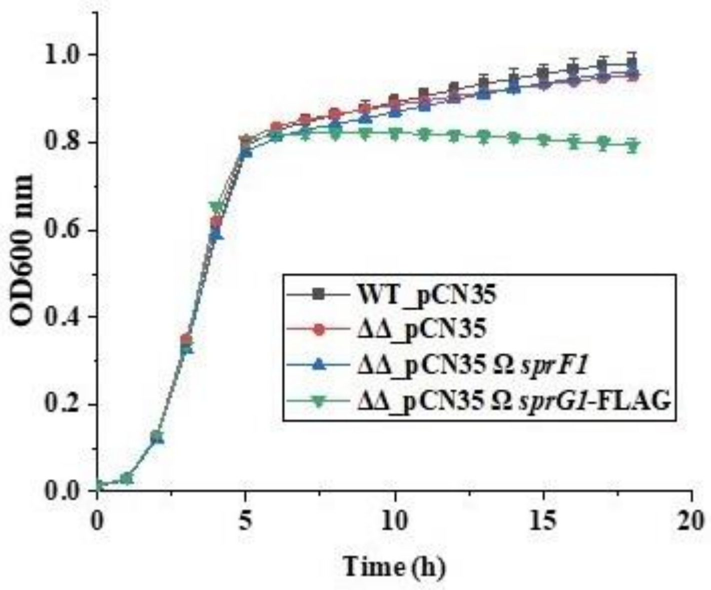

Supplementary Figure S3. Growth curves of *S. aureus* N315 (WT) and the *sprG1/sprF1* deletion mutant (ΔΔ) transformed with the control plasmid (pCN35), the plasmid (pCN35Ω*sprF1*) expressing SprF1 antitoxin under the control of its native promoter or the plasmid (pCN35Ω*sprG1*-FLAG) expressing the flagged version of SprG1 toxin, cultivated in optimal conditions (Tryptic Soy Broth, 37 °C)

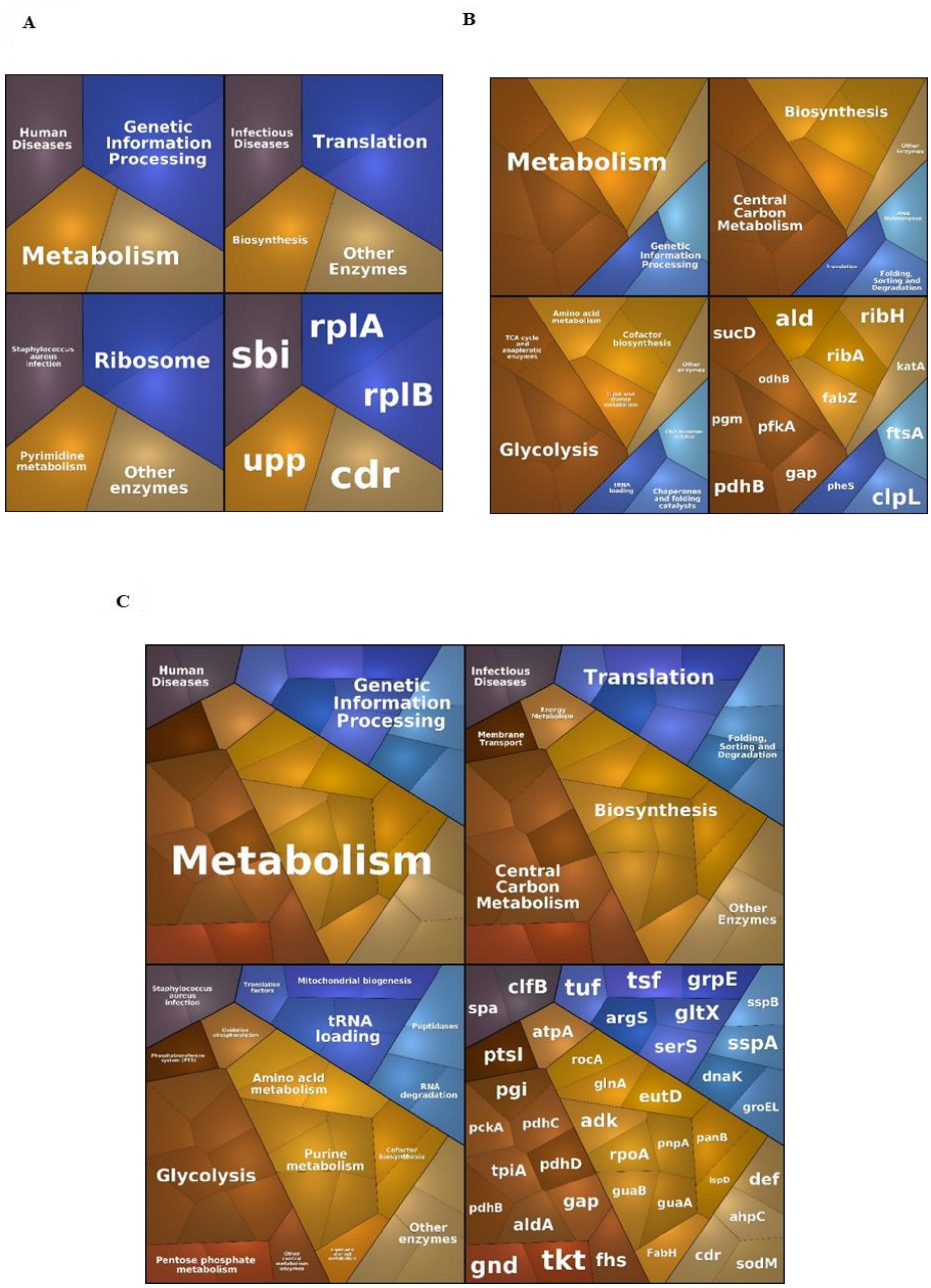

Supplementary Figure S4. Voronoi diagrams illustrating functions of differentially expressed proteins identified in proteomic studies. (A) and (B) intracellular proteomes at logarithmic and stationary growth phases, respectively. (C) The extracellular proteome at stationary phase. The diagrams were prepared using interactive proteomaps (available at www.proteomaps.net; Liebermeister W, Noor E, Flamholz A, Davidi D, Bernhardt J, and Milo R, Visual account of protein investment in cellular functions. PNAS June 10, 2014 111 (23) 8488-8493).

Supplementary Table S1. A list of differentially expressed proteins extracted from proteomic comparisons of *S. aureus* N315 with its deletion mutant of SprG1/SprF1 TA system and with the (over)expression of SprF1 antitoxin – see the excel file: Supplementary Table_1.xlsx

Supplementary Table S2. SprF1 (sRNA) interactions with *S. aureus* N315 gene transcript predicted by the TargetRNA2 algorithm (TargetRNA2: identifying targets of small regulatory RNAs in bacteria. Mary Beth Kery, Monica Feldman, Jonathan Livny, and Brian Tjaden. Nucleic Acids Research, 42(W1): W124-W129, 2014).

Rank	Gene	Synonym	Energy	P_values	sRNA_start	sRNA_stop	mRNA_start	mRNA_stop
1	-	SA0971	-18.24	0.000	13	27	-76	-62
2	*lytS*	SA0250	-13.49	0.003	120	133	2	15
3	-	SA0270	-13.26	0.003	88	105	-17	2
4	-	SA2179	-11.20	0.012	59	78	-3	17
5	-	SA0517	-10.77	0.016	61	75	9	20
6	*aldH*	SA1736	-10.37	0.019	41	55	-72	-60
7	*cfxE*	SA1065	-10.00	0.023	21	29	-72	-64
8	*set12*	SA0388	-9.93	0.024	10	19	-79	-70
9	*acpS*	SA1875	-9.75	0.026	96	109	-55	-42
10	*asnC*	SA1287	-9.39	0.031	90	105	-12	3
11	*menE*	SA1615	-9.36	0.032	122	136	-68	-54
12	*splF*	SA1627	-9.11	0.035	127	141	6	20
13	-	SA0524	-9.03	0.037	49	64	-64	-49
14	-	SA1764	-9.01	0.037	94	109	-48	-33
15	-	SA2123	-8.98	0.038	128	141	-73	-60
16	-	SA0224	-8.88	0.039	59	74	-69	-54
17	-	SA0815	-8.81	0.040	119	130	-1	11
18	-	SA0116	-8.68	0.043	120	134	-63	-49
19	-	SA0286	-8.43	0.047	29	41	-11	4
20	*ddh*	SA2312	-8.36	0.049	72	86	7	20
21	-	SA0860	-8.34	0.049	111	119	-21	-13
22	-	SA0268	-8.32	0.049	95	108	-11	4
23	-	SA1778	-8.30	0.050	126	137	-28	-17

```
SA0971  (conserved hypothetical protein)
        sRNA    27      3' ACGCGUACAACGGGA 5'  13
                            ||||||||||::|
        SA0971  -76     5' CGCGCAUGUUGUUCA 3'  -62

lytS (Sensor histidine kinase/phosphatase LytS)

        sRNA    133     3' UUGGUAGCGAUUGA 5'   120
                            :|:|||||||||
        lytS    2       5' UGCUAUCGCUAACA 3'   15

SA0270 (similar to secretory antigen precursor SsaA)

        sRNA    105     3' ACC-AAUUUACCAAUAAUU 5'      88
                            || ||||||||||||||
        SA0270  -17     5' AGGAUUAAAUGGUUAUUAU 3'      2

SA2179 (Oxygen regulatory protein NreC)
```

```
        sRNA    78      3' AUUAGAUUUUAUCGGUGGCA 5'     59
                            |||: |||||||:||::|
        SA2179  -3      5' AAAUUGAAAAUAGUCAUUGC 3'     17
```

SA0517 (Cof-type HAD-IIB family hydrolase)

```
        sRNA    75      3' AGAUUUUAUCGGUGG 5'  61
                            ||  |||||||| |
        SA0517  9       5' ACU--AAUAGCCA-C 3'  20
```

aldH (Aldehyde dehydrogenase)

```
        sRNA    55      3' AAAUUGCCCGAGUGA 5'  41
                            ||||| ||| |||
        aldH    -72     5' AUUAAC-GGC-CACA 3'  -60
```

cfxE (Ribulose-5-phosphate 3-epimerase homolog)

```
        sRNA    29      3' AAACGCGUA 5'21
                            |||||||
        cfxE    -72     5' AUUGCGCAA 3'-64
```

set12 (Superantigen-like protein SSL8)

```
        sRNA    19      3' AACGGGAAAA 5'       10
                            ||||||||
        set12   -79     5' AUGCCCUUUA 3'       -70
```

acpS (Holo-[acyl-carrier-protein] synthase)

```
        sRNA    109     3' AGCUACCAAUUUAC 5'   96
                            ||||||||||||
        acpS    -55     5' ACGAUGGUUAAAUA 3'   -42
```

asnC (Asparagine--tRNA ligase)

```
        sRNA    105     3' ACCAAUUUACCAAUAA 5' 90
                            ||  :|||||||||
        asnC    -12     5' AGG-AGAAUGGUUAUG 3' 3
```

menE (2-succinylbenzoate--CoA ligase)

```
                         11
        sRNA    136     3' AUAUUGGUAGCGAUU 5'  122
                            ||||||| :||||:
        menE    -68     5' CAUAACCACUGCUAG 3'  -54
```

splF (Serine protease SplF)

```
        sRNA    141     3' UUUUUAUAUUGGUAG 5'  127
                           ||||||||||:|||
        splF    6       5' AAAAAUAUAAUCAU- 3'  20
```

SA0524 (GTP cyclohydrolase)

```
        sRNA    64      3' GUGGCAGAAAAAUUGC 5' 49
                           ||| | |||||||||
        SA0524  -64     5' CACAG-CUUUUUAACA 3' -49
```

SA1764 (Phage minor structural protein)

```
        sRNA    109     3' AGCUACCAAUUUACCA 5' 94
                            ||| ||||||||||
        SA1764  -48     5' ACGACGGUUAAAUGGC 3' -33

SA2123 (Transcription regulator LysR family)

        sRNA    141     3' UUUUUAUAUUGGUA 5'   128
                            ||||||||:|||
        SA2123  -73     5' UAAAAUAUAGCCAA 3'   -60

SA0224 (6-phosphogluconate dehydrogenase, decarboxylating)

        sRNA    74      3' GAUUUUAUCGGUGGC-A 5'        59
                            |||||||| || || |
        SA0224  -69     5' AUAAAAUAG-CAACGUU 3'        -54

SA0815 (Peptidyl-prolyl cis-trans isomerase, PPIase)

        sRNA    130     3' GUAGCGAUUGAA 5'     119
                            || |||||||
        SA0815  -1      5' AAUGGCUAACUA 3'     11

SA0116 (Siderophore biosynthesis protein SbnE)

        sRNA    134     3' AUUGGUAGCGAUUGA 5'  120
                            ||||||| ||||:
        SA0116  -63     5' CAACCAUCACUAAUC 3'  -49

SA0286 (TIGR01741 family protein)

        sRNA    41      3' AUCC-C-AUUGUACA 5'  29
                            :|| | |||||||
        SA0286  -11     5' GGGGCGAUAACAUGA 3'  4

ddh (D-lactate dehydrogenase)

        sRNA    86      3' AAUUAGAAAUUAGAU 5'  72
                            |||||||||||:|
        ddh     7       5' AUAAUCUUUAAUUU- 3'  20

SA0860 (ClpXP adapter protein SpxH)

        sRNA    137     3' UAUAUUGGUAGC 5'     126
                            |||||||||:
        SA1778  -28     5' UUAUAACCAUUU 3'     -17
```

Parameters for TargetRNA2 algorithm (http://cs.wellesley.edu/~btjaden/TargetRNA2/index.html):

TargetRNA version:	2.01
Replicon:	*Staphylococcus aureus* subsp. aureus N315 chromosome
NTs before start codon:	80
NTs after start codon:	20

Seed length:	7
sRNA conservation and accessibility:	true
sRNA window size:	13
mRNA structural accessibility:	true
Interaction region:	20
Filter size:	400
Single target:	-
File of candidate targets:	-
p-value:	0.05

sRNA: SprF1 RNA antisense

```
>SprF1
AUAUAUAGAAAAAGGGCAACAUGCGCAAACAUGUUACCCUAGUGAGCCCGUUAAAAAGACGGUGGCU
AUUUUAGAUUAAAGAUUAAAUUAAUAACCAUUUAACCAUCGAAACCAGCCAAAGUUAGCGAUGGUUA
UAUUUUU
```
